# Quinone binding in respiratory complex I: Going through the eye of a needle. The squeeze-in mechanism of passing the narrow entrance of the quinone site

**DOI:** 10.1007/s43630-021-00113-y

**Published:** 2021-11-23

**Authors:** Nithin Dhananjayan, Panyue Wang, Igor Leontyev, Alexei A. Stuchebrukhov

**Affiliations:** Department of Chemistry, University of California at Davis, One Shields Avenue, Davis, CA 95616 USA

**Keywords:** Complex I, Ubiquinone, Electron transport chain

## Abstract

At the joint between the membrane and hydrophilic arms of the enzyme, the structure of the respiratory complex I reveals a tunnel-like Q-chamber for ubiquinone binding and reduction. The narrow entrance of the quinone chamber located in ND1 subunit forms a bottleneck (eye of a needle) which in all resolved structures was shown to be too small for a bulky quinone to pass through, and it was suggested that a conformational change is required to open the channel. The closed bottleneck appears to be a well-established feature of all structures reported so-far, both for the so-called open and closed states of the enzyme, with no indication of a stable open state of the bottleneck. We propose a squeeze-in mechanism of the bottleneck passage, where dynamic thermal conformational fluctuations allow quinone to get in and out. Here, using molecular dynamics simulations of the bacterial enzyme, we have identified collective conformational changes that open the quinone chamber bottleneck. The model predicts a significant reduction—due to a need for a rare opening of the bottleneck—of the effective bi-molecular rate constant, in line with the available kinetic data. We discuss possible reasons for such a tight control of the quinone passage into the binding chamber and mechanistic consequences for the quinone two-electron reduction.

**Graphic abstract:**

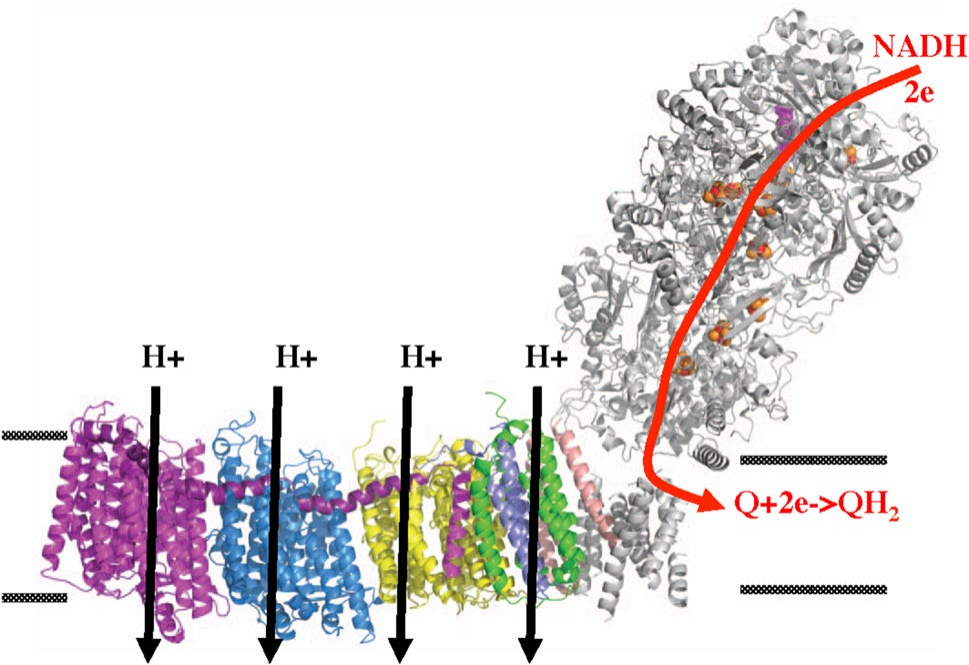

**Supplementary Information:**

The online version contains supplementary material available at 10.1007/s43630-021-00113-y.

## Introduction

NADH:ubiquinone oxidoreductase, or respiratory complex I, is a key proton-pumping enzyme of the energy-generating machinery in the cell [1, 2]. Recent structural studies [3–13] of the enzyme have revealed molecular details that suggest possible molecular mechanisms of its redox-driven proton pumping. Complex I is an L-shaped structure with a hydrophilic domain where electron transport takes place and a membrane domain that performs proton translocation [4, 14]. In the hydrophilic domain, NADH transfers 2 electrons to flavin mononucleotide (FMN), which then transfers electrons via a chain of seven iron sulfur (FeS) clusters to a quinone molecule (*Q*) reducing it to a quinol [15]. The transfer of two electrons to quinone [16] is a key exergonic step, which is believed to drive local conformational changes [2, 11, 17] that transmit to the membrane domain of the complex and help to drive the proton pumping [18, 19]. The new structures have also opened a new intriguing question about the mechanism of quinone binding to the enzyme, which is addressed in this paper.

In all organisms, from bacteria to human, the structure of the core part of the enzyme reveals an almost 30 angstrom tunnel-like chamber for ubiquinone binding (Q-chamber) that leads from the N-edge of the membrane up to N2 FeS cluster. Presumably, the quinone molecule migrates from the membrane into the binding chamber, diffuses up to N2 cluster, receives two electrons and migrates back – tail first, from the narrow binding cavity to the membrane [8, 18, 20–25].

In all organisms, the core part of the enzyme is very similar to bacterial enzyme; here, the entrance to Q-chamber is formed by a specific crossing of TM1, AH1, and TM6 in Nqo8 (mtND1/H *E.coli*) [4] and forms a narrow bottleneck that restricts the access to the Q-tunnel. The bottleneck was identified in the structure early on [4, 26], and it was speculated that conformational changes are needed to open it; however, the molecular mechanism of quinone passing the narrow bottleneck remains to be obscure, see Ref. [27] Recently [28], we characterized the entrance bottleneck more rigorously using Molecular Dynamics (MD) simulations to quantify the energy barrier formed by the narrow bottleneck. Computer simulations of quinone passage through the bottleneck suggest that in all structures available, from bacterial to human (including most recent structures [3, 11–13], see below), this bottleneck is too narrow for the quinone or quinol to pass and that a conformational change is indeed required to open the channel. Moreover, in yeast *Y. lipolytica *[8] structure, the quinone is seen bound in the cavity, with a half of the isoprenoid tail crossing the bottleneck. However, here too the bottleneck (taken as in the reported pdb structure) was found to be too narrow for the head group, or even for the isoprenoid tail, to freely move through the narrow entrance of the quinone chamber, indicating the quinone molecule is stuck in the position seen in the structure and suggesting that dynamic or static opening of the bottleneck is needed to allow movement of the quinone. Thus, the question arises as to how the bulky substrate is going through a narrow passage, as if through the eye of a needle?

Previously we concluded that the apparent bottleneck closed structure could be explained by two possibilities: in one, the closed structure is an artifact of the crystallization packing forces, cryo-EM low temperature, or other specific conditions occurring in the structural data acquisition that affect this flexible part of the enzyme, assuming that a functional open bottleneck structure exists in the natural membrane environment of the enzyme, yet unseen in the available structures. Another possibility is that the stable open bottleneck state in enzyme does not actually exist, and only rare thermal fluctuations of the enzyme structure would open the bottleneck and allow admission of the quinone molecule to the quinone chamber, with tightly controlled overall passage of the quinone to the binding site by some intricate mechanism.

Most recent data [3, 11] indicate that in all available structures (twenty-three analyzed so far) including both so-called “open” and “closed” states [3, 11] (not to be confused with open and closed bottleneck of this paper), involving well-resolved structural changes of other parts of the enzyme seen both in X-ray and cryo-EM, the bottleneck is about the same and is in the closed state as we identified it. Thus, the new data suggest that the stable open bottleneck state (or quasi-stable state with significant thermal population to be captured in the plunge-freezing of cryo-EM) does not seem to exist. Therefore, it now appears more likely that the bottleneck opening occurs dynamically in the course of thermal fluctuations, suggesting a specific intricate mechanism of the passage of the quinone through the bottleneck.

Here, using molecular dynamics simulations (both all-atomic and coarse grained) of the bacterial enzyme we have identified collective conformational changes (Principal Component Analysis, PCA, modes) that dynamically open the quinone chamber bottleneck. The changes involve mostly TM1 helix, which straightens up, AH1 helix, which moves to open the structure, and the loop between them; but in general, the changes involve rearrangement of a larger part of the enzyme. We propose a specific “squeeze-in” mechanism of the bottleneck passage, where the rare conformational fluctuations along the identified PCA mode allow quinone in and out.

The simulations indicate that the most flexible part of the enzyme Nqo8 subunit involves structural elements that form the bottleneck; this suggests that if external forces—e.g., from the bound adjacent subunits—were applied, the entrance into Q-chamber would be affected most, possibly to be squeezed and locked in a closed state. This could explain the closed bottleneck state in all the resolved structures. The model predicts a significant reduction—due to a need for a rare opening of the bottleneck—of the effective bi-molecular binding rate constant, which is in line with the available kinetic data. We discuss possible reasons for such a tight control of the passing of the quinone into the binding chamber, which remain to be obscure at present.

## Methods and results

### The Bottleneck of Q-chamber

Previously [28], five structures were analyzed: bacterial *T. thermophilus *[4], yeast *Y. lipolytica *[8], and three mammalian structures, ovine [5], mice [9], and human [10, 29]. As the core part of the enzyme is almost identical to the 14 subunits of the bacterial complex, and the results are qualitatively similar for all structures analyzed, here we consider first the bacterial structure. The entrance of the quinone chamber is formed in ND1 subunit (Nqo8/H subunit of bacterial enzyme); the elastic properties of this subunit, considered within the enzyme context, define the static and dynamic properties of the bottleneck.

**Fig. 1:**
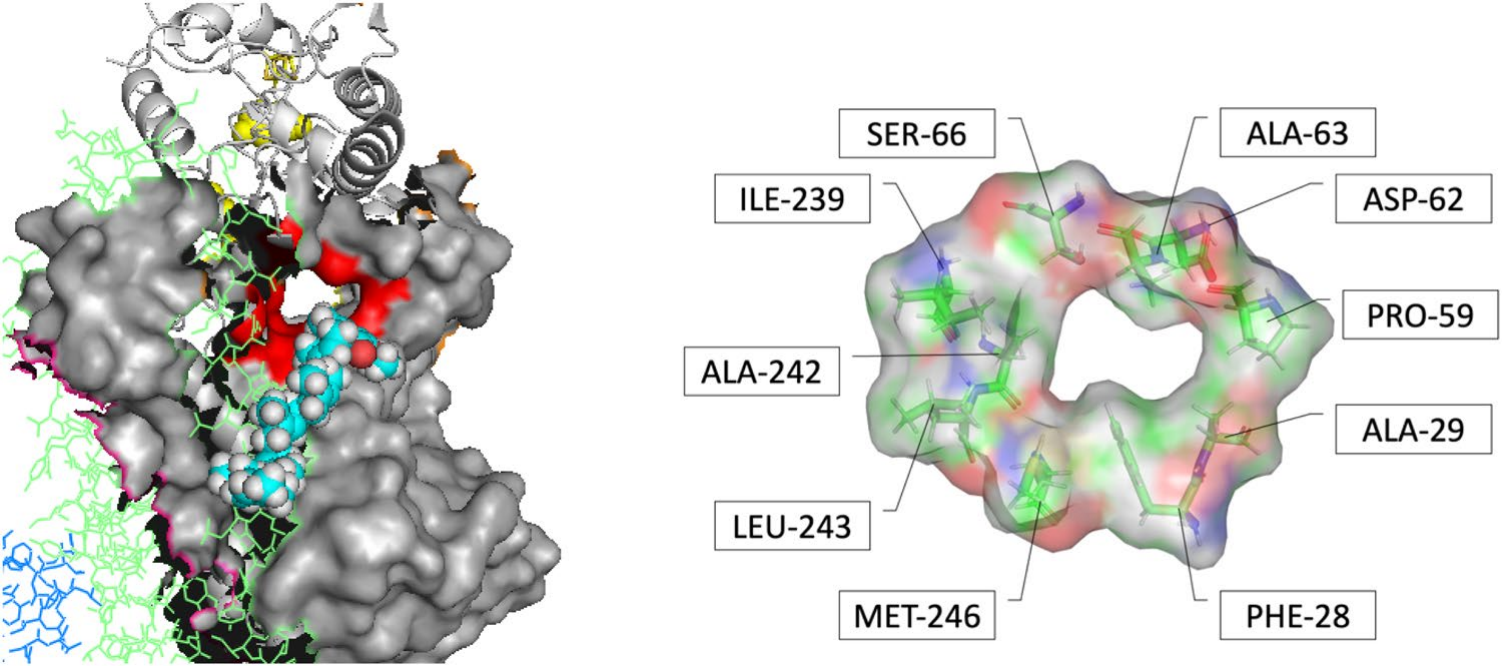
**A** Left. The entrance into the Q-binding cavity is shown in red. Gray surface refers to subunit ND1, green lines—subunit A, and cyan spheres represent the quinone (from MD simulations in Ref. [28]). **B** Right. 10 residues that form the entrance of complex I Q-cavity in bacterial enzyme; the bottleneck is in the opening in the middle of the structure

For the analysis of the bacterial complex I, we use the structure *T. thermophilus,* 4HEA [4] with the highest resolution of 3.1 Å. The entrance to quinone binding cavity and the amino acids comprising the orifice of the entrance, i.e. the bottleneck, are shown in Fig. 1.

The bottleneck is localized at the narrow crossing framed by three helices: TM1, AH1, and TM6 [4] of ND1/Nqo8 subunit (see below); the properties of the bottleneck is the focus of the MD simulations in this paper.

### The bottleneck is about the same in all structures available

**Fig. 2:**
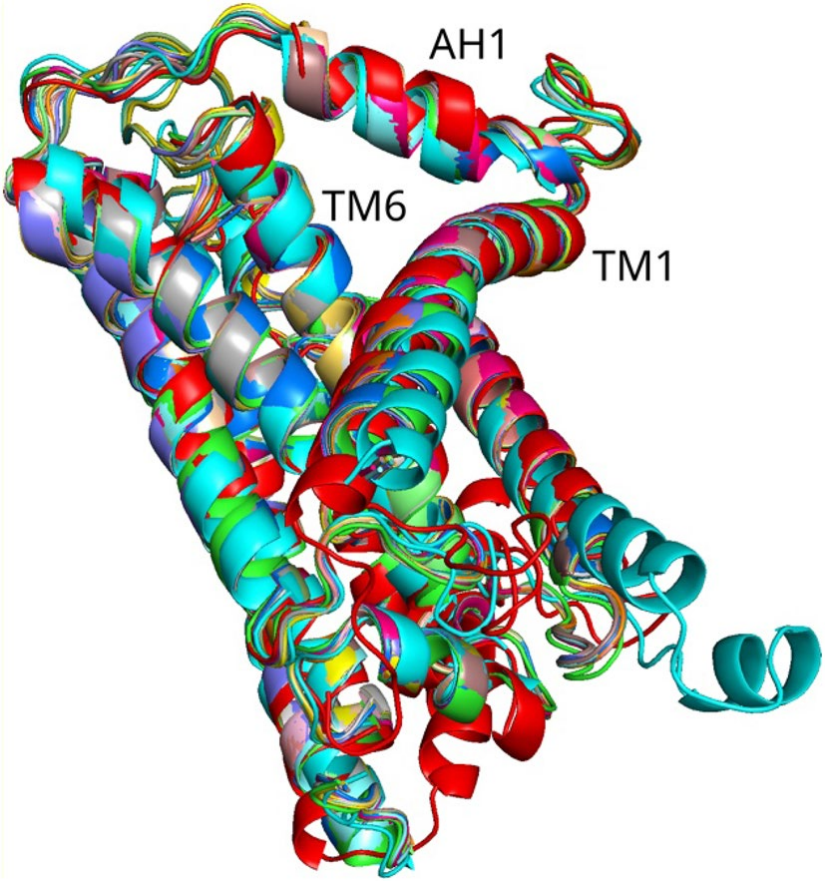
Overlap of all 23 chain-H structures. The bottleneck is formed by the crossing of three helices: TM1, AH1, and TM6 of ND1/H/Nqo8 subunit. The crystal structure of bacterial (*T. thermophilus*) is shown in red and appears to be the most open among all structures analyzed. The shown structures include different organisms and different so-called “open” and “closed” states of the enzyme [3, 11]

Recently, the range of the available structures has been significantly expanded [3, 11–13]; several structures, both X-ray and cryo-EM, now indicate different conformational states that the enzyme assumes during its catalytic cycle [3, 11]. Presumably, the structures capture quasi-stable states, with significant thermal population fraction that reflect different conformations; for example, in some structures the angle between the membrane and the peripheral arms changes as much as 6°–7°, or significant changes are observed inside of the Q-binding cavity. Yet, the bottleneck in all structures available (we analyzed twenty-three such structures) are about the same, as shown in Fig. 2.

### The bottleneck is closed in all resolved structures

We showed previously that taken as in the pdb structure, the bottleneck is essentially closed. We demonstrate this by calculating the barrier of crossing the bottleneck structure. This is done in the following manner. (Additional probes were explored in Ref. [28]).

#### Barrier simulations

The MD simulation details are given in SI and are the same as in Ref. [28] Briefly, the protein was incorporated into POPC membrane. The quinone was placed near the entrance of Complex I, and after equilibration, was pulled into quinone cavity. We also pulled quinone out of the quinone cavity. The energy and the pulling force were measured along the pulling trajectory. Both ubiquinone and menaquinone with various tail lengths were simulated, see details in Ref. [28]. To improve statistics, focused MD simulations on a restricted system that involved only the residues of the bottleneck (Fig. 1) were used. (For additional simulation details, see MD Methods in SI.)

**Fig. 3:**
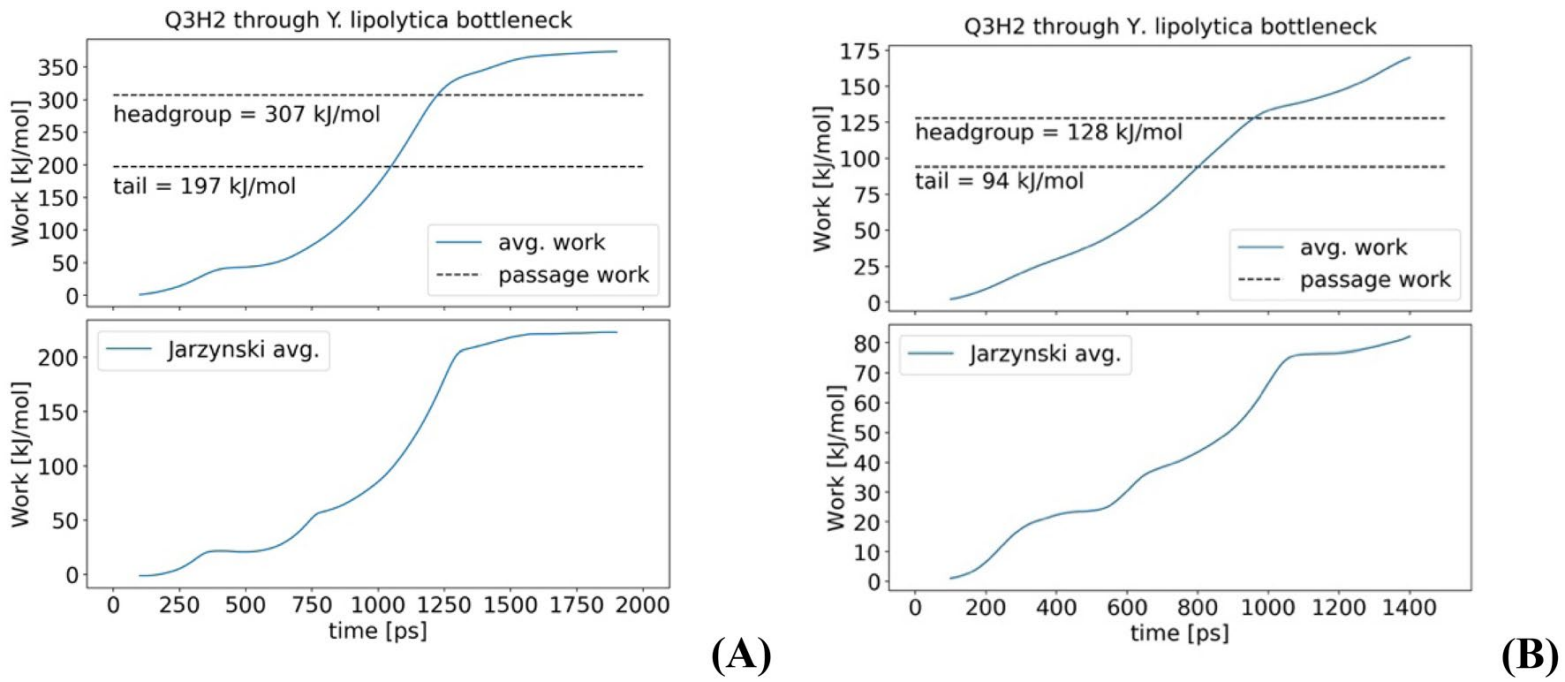
The average work along the pulling trajectory of ubiquinone (reduced, Q_3_H_2_, with 3 isoprenoid units) through the bottleneck of the Q-chamber in *Y. lipolytica* enzyme. The values under dotted lines give corresponding energy barriers for the headgroup and the two isoprenoid units of the tail passage. Lower panels show JA work along the pulling trajectory. **A** All atoms are strongly restrained as in pdb structure. **B** Backbone atoms are weakly restrained by 50 kJ mol^−1^ Å^−2^, and side-chain atoms by 10 kJ mol^−1^ Å^−2^ (a hydrogen bond, for comparison, is about 20 kJ/mol)

Using pulling trajectories, we calculated the Helmholtz work (or Helmholtz free energy,$W$) to cross the bottleneck. In addition, we calculated so-called Jarzynski Averaged (JA) work [30]. Here the conditions of simulation are different from what is assumed in the JA work; however, we find it useful to consider this type of averaging as it emphasizes trajectories with minimal work and thus selects (or filter out) the “optimal” trajectories. As such, JA is sensitive to qualitative changes along the trajectory, such as the entrance of the headgroup into the bottleneck or the passage of individual methyl groups of isoprenoid tail of ubiquinone, which show up as small bumps on the JA curves, see Fig. 3 below.

Here the goal was to probe the bottleneck passage in the structures given directly by the reported X-ray or cryo-EM pdb and to see if conformational changes are needed for the passage. Therefore, the backbone atoms were restrained to the positions given by the pdb structures, and different strengths of restraint were probed. Asp of the bottleneck was protonated in our simulations. Typical results are shown in the following figure.

Figure 3 shows a typical example of the average work of bottleneck crossing by the reduced quinone, Q_3_H_2_, in yeast *Y. lipolytica* enzyme. (Most simulations were done with 3-isoprenoid unit tail ubiquinone, half-inserted in the Q-chamber; due to repetition of the tail structure, the Q_10_ results could be inferred from the data.) Here the cryo-EM structure gives the initial position of the quinol (reduced) in the Q-cavity. We calculate the barrier to move the quinone in the structure captured by cryo-EM. The dotted lines in the figure correspond to so-called “first passage” work, i.e. work required to find and enter the bottleneck by the headgroup of QH_2_ and moving the methyl groups of the tail through the narrow entrance. In this case, two methyl groups were passing the bottleneck. The reduced form of QH_2_ requires some 20 kJ/mol more energy to pass the bottleneck barrier. The found average work is obviously too high to pass the bottleneck without conformational opening of the structure.

As can be seen, the barriers are too high for the bottleneck passage both for the headgroup and for the tail. The weaker restraints on the structure were also probed, Ref. [28], but the barriers still remained too high to allow a suitable timescale of passage. A reasonable barrier that would give a *ms* timescale should produce a barrier no higher than some 30 kJ/mol, see Discussion.

The results for all other enzymes, including bacterial, mt ovine, and human enzymes, yield the same qualitative conclusion—the bottleneck is too narrow for the headgroup and even for the tail passage (yeast), as the barrier to cross the bottleneck is too high.

We conclude that as seen in the resolved structures, the size of the entrance is prohibitively small for the quinone molecule to pass either in oxidized or in the reduced form. There are slight variations in all structures examined, but in all structures the bottleneck is too narrow to be operational to admit quinone to the binding site. There are two possibilities: one is that quinone may never get out of the quinone cavity and works as an electron shuttle, another is that dynamic conformational changes open the bottleneck and allow quinone in and out. After exploring details of conformational dynamics below, we will discuss both possibilities in the last section of the paper.

### Bottleneck opening in ND1 subunit

We now turn to exploring possible conformational openings of the bottleneck in ND1 subunit (without quinone, assuming binding by an empty enzyme). The idea is to first use the most accurate all-atomic force field and explore the elastic properties of isolated ND1 subunit itself; we then extend simulations to include other subunits adjacent to ND1 using a less accurate coarse-grained force field.

#### MD simulations

In MD simulations, we use Gromacs [31] simulation package with CHARMM36 forcefield [32]. The initial coordinates of ND1 (Nqo8) subunit were extracted from the whole structure of *T. thermophilus* [4], and ND1 was simulated as described in MD Methods of SI. The Principal Component Analysis detailed below was applied to analyze the trajectories.

#### Principal component analysis (PCA) of MD trajectories

The PCA method is described in Ref. [33]. Here, we briefly summarize our approach.

The PCA normal modes are collective coordinates $Q_{1} ,Q_{2} , \ldots$ that are linear combinations of the usual (mass-weighted) Cartesian displacements of the protein atoms $x_{i}$ from their average positions:

$$Q_{\lambda } = \sum {x_{i} S_{i\lambda } } ,$$

where $i = (a,\sigma )$, *a* – atom/site number, $\sigma = x,y,z$. The expansion coefficients $S_{i\lambda }$ are found by diagonalization of the correlation matrix $M_{ij} = < x_{i} x_{j} >$, where averaging < … > is assumed along the MD trajectory. After diagonalization, the diagonal elements of the correlation matrix give PC variances – the averaged values of squared amplitudes of PCA modes:

$$(diagM)_{\lambda \mu } = \delta_{\lambda \mu } < Q_{\lambda }^{2} > .$$

The expansion coefficients $S_{i\lambda }$ are eigenvectors of the correlation matix. The square values $P_{i\lambda } = \left( {S_{i\lambda } } \right)^{2}$ can be considered as “probabilities” (after proper normalization) and $S_{i\lambda }$ as “amplitudes” for a given eigenvector. Atom participation value is defined by summing $P_{i\lambda }$ for a given atom *a* over *x, y, z* components. The PCA modes are similar and formally equivalent to familiar normal modes of an artificial harmonic system with the same displacement correlation matrix.

Each normal mode can be thought as describing coherent motion of all coordinates *x* involved in it:

$$\begin{gathered} x_{i} (t) = S_{i\lambda } \cdot Q_{\lambda } \hfill \\ Q_{\lambda } = Q_{\lambda 0} \cos \left( {\omega_{\lambda } t} \right) \hfill \\ \end{gathered}$$

The picture of coherent motion is only qualitative because there is significant dumping of the oscillations and the low-frequency modes are mostly in the over-dumped regime. However, the picture of normal modes is a convenient way to think about the collective or correlated motions of a big system.

The frequency of each mode can be found from

$$\omega_{\lambda } = \sqrt {\frac{{k_{\lambda } }}{{M_{\lambda } }}} .$$

Here the force constant $k_{\lambda }$ and the effective mass of a mode can be found from the energy relations. From the averaged potential energy,

$$k_{\lambda } = \frac{{k_{B} T}}{{ < Q_{\lambda }^{2} > }},$$

and from kinetic energy ($m_{i}$ are atomic masses):

$$M_{\lambda } = \sum {m_{i} S_{i\lambda }^{2} } .$$

When the mass-weighted coordinates are used, the effective masses are all the same and can be normalized to unity. The timescales of the low-frequency modes (periods $T_{\lambda } = 2\pi /\omega_{\lambda }$) can be large and therefore difficult to access via direct MD. One can use these modes for probing possible large amplitude motions of the protein by artificially moving along one or several low-frequency modes, guiding direct MD, and thus more efficiently sample phase space. Also, it was suggested [34] that the functional or essential conformational changes of proteins most likely occur along with the low-frequency collective coordinates, as a low energy path for large change.

## Results

### 1. Bottleneck opening is related to soft collective modes of ND1

**Fig. 4:**
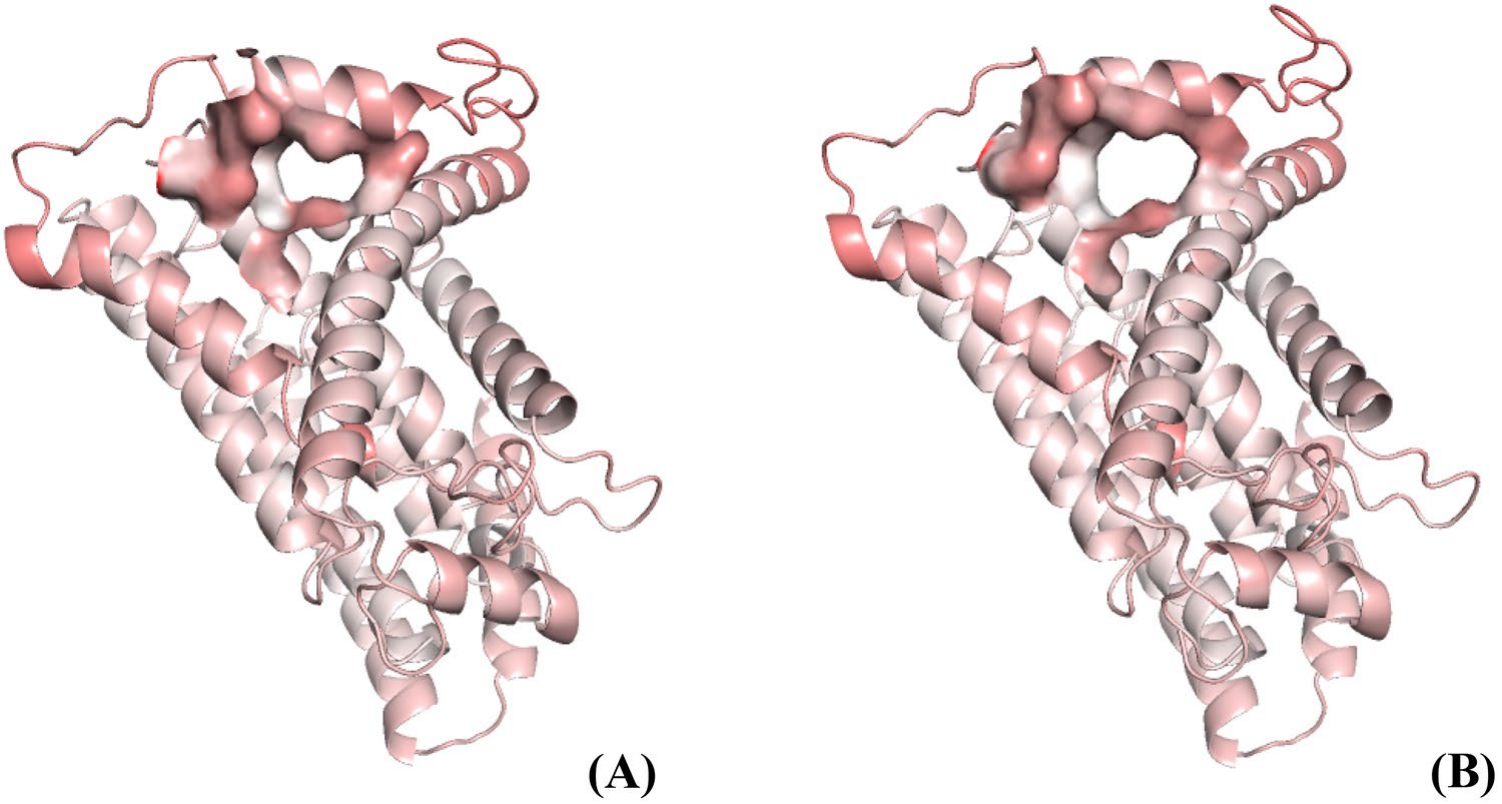
The helix structure representation of H-subunit (ND1) of complex I and the entrance into Q-binding cavity. **A** Left, closed structure; **B** Right, open bottleneck structure. The opening occurs in deformation along the lowest frequency collective PCA mode (Q0), see Movie S1 in SI. The red color represents atoms and residues that contribute most to PCA mode Q0. The un-bending deformation of TM1 helix and the increased size of the bottleneck are two most prominent features of Q0. Details of several other modes are discussed in the text, and further details are given in SI

The Principal Component Analysis of an MD trajectory results in several collective modes $Q_{\lambda }$, which are graded and ordered by the magnitute of their elasticity-strength constant $k_{\lambda }$ and frequency $\omega_{\lambda }$; several such modes are listed in Table S1–S4 in SI. Different modes involve different types of collective motions (see selected mode animations in SI) and represent different types of collective deformations under external stress on the protein structure. Surprisingly, we find the very first mode, with the lowest elastic constant involve structural elements of the bottleneck; namely, helices TM1, AH1, and TM2 and the two loops connecting them, as seen in Fig. 4 and corresponding animation, Movie S1, in SI. The involvement (or participation value) of specific amino acids in a given PCA mode is found as described in the Methods section, and the structural elements with the highest participation are represented by the color intensity in Fig. 4 (see also related figures and animations in SI). The animation of this mode opening the bottleneck is shown in Movie S1 in SI.

It is seen in Fig. 4 and Movie S1 in SI that the deformation along with the first PCA mode Q0 mainly involves helices TM1, AH1, and TM2 and the two loops connecting them. The un-bending deformation of TM1 helix and the increased size of the bottleneck are the two most prominent features of the softest deformation mode of ND1. This mode also involves the opening of the so-called E-channel in H/ND1-subunit, see S1 in SI. The timescale of this collective motion is 0.3 ns, Table S1; this is a slow motion, about thousand times slower than the usual molecular vibrations (e.g., CC bond). Details of several other modes are discussed later in the text, and further details are given in SI. Generally, we find that many low-frequency modes involve the motion of TM1 and AH1 helices, but their contribution is scaled by their amplitudes—the higher the frequency the lower the amplitude, and so is the contribution.

As the structural elements of the bottleneck—TM1, AH1, and TM6—correspond to the softest mode, it is clear that if external forces were applied to deform ND1 subunit, the deformation would firstly affect the bottleneck structure. Thus, the closure of the bottleneck can be expected if one assumes compressing, de-solvation forces acting on the protein in the structure-resolution conditions or low temperature, which is particularly clearly seen in Movie S1 in SI.

### Bottleneck participation spectra

To evaluate quantitatively the involvement of the bottleneck residues in a given collective mode, we calculate the so-called participation value; namely, for the bottleneck residues, A29, F28, P59, D62, A63, S66, I239, A242, L243, and M246, shown in Fig. 1, we calculate the total participation probability of all atoms involved as ${I}_{\lambda }=\sum_{a\epsilon {\text{bottleneck}}}{P}_{a\lambda }$. The resulting values then are scaled according to thermal amplitudes of the modes (eigenvalues of the PCA) and normalized to unity. Each mode thus is assigned the total bottleneck participation value, by which its contribution to the bottleneck conformations can be evaluated. For a group of modes, these data provide a spectrum of bottleneck participation. Alternatively, we directly calculate the variation of the size of the bottleneck (measured by its shortest dimension) for each mode, assuming the average thermal amplitudes of the modes.

**Fig. 5:**
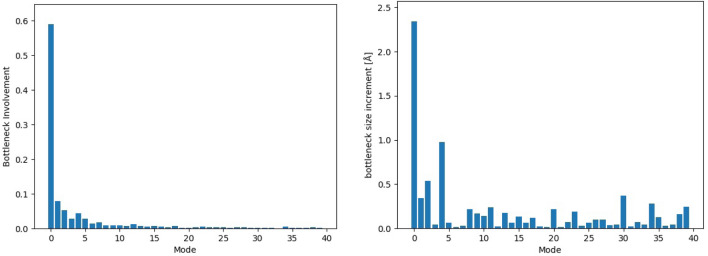
Left: Bottleneck involvement in the first 40 modes. The higher modes are increasingly stiffer in character with less relevance to large-scale structural changes. Right: Bottleneck size increments for the first 40 modes

Figure 5 shows the spectrum of the first 40 (out of more than 8000) modes. It is clear that the first few low-frequency modes are most important, based both on formal participation and in their variation of the bottleneck size. In SI (S2–S4) several animations of typical modes with high participation in the bottleneck are shown.

It is of interest to characterize the qualitative differences between different modes. If we neglect the difference in amplitudes and only focus on how coordinates are mixed in a collective mode (see Fig. S1), Modes 0, 4 and 5 show a higher bottleneck involvement than the rest of the first 10 low-frequency modes. Those modes are shown in animations in SI, Movies S1a-b. These examples clearly illustrate the different characters of the collective motions involved; surprisingly, many of them involve some elements of the bottleneck. We need to remember, however, that participation of different modes is scaled by their thermal amplitudes, and thus in practice we can focus only on a few first low-frequency modes, with the first mode clearly dominating, Fig. 5, left panel.

Indeed, the first mode Q0 shown in Fig. 4 already tells most of the story; namely, it is TM1 straightening and the related upside AH1 motion that are mostly responsible for the opening of the bottleneck. For a system involving tens of thousands of coordinates, to find only a few functionally relevant collective coordinates is quite remarkable. In the following section, we will explore how adjacent subunits affect this conclusion.

### The structure of the open bottleneck state

An open bottleneck structure under Q0 deformation, see Fig. 4 and animation S1 in SI, can be envisioned as a possible open state of the bottleneck. On average, the changes are relatively small, but they involve rearrangement of essentially the whole subunit ND1 (as is particularly clearly seen in all-atom animation of Q0 in SI). As this subunit is in contact with other parts of the enzyme, including membrane subunit A (ND3), some changes in the equilibrium structure of the whole membrane part are expected in the open functional state. It is interesting that deformation under Q0 mode also opens the so-called E-channel in the ND1 subunit, see SI, Movie. S1C. In simulations, the open state (maximum amplitude of Q0 quasi-harmonic deformation) occurs only in the course of thermal fluctuations, i.e. it is not a stable state. This is in line with most recent structural data where no open state was detected, see Fig. 2; thus, the bottleneck open state occurs as a rare thermal fluctuation.

### Stressed conditions of ND1 subunit in the enzyme structure

By design, in this section our simulations involved isolated ND1 subunit with no specific external forces acting on it (except for hydrophobic solvent—to model the inner part of the membrane, external pressure from the surrounding environment, including adjacent subunits, and some restriction of the terminal residues. This is still not an accurate representation of the enzyme context, which is examined in greater detail in coarse-grained simulations later.) This is done in part to explore the effect of the stress conditions [35] on the subunit when it is part of the whole enzyme structure; the stress conditions are due to inter-subunit forces, proper membrane solvation [35], etc. To evaluate the effect of boundary conditions imposed by the neighboring subunits, here we artificially remove the external forces that keep ND1 subunit in the constrained configuration in the enzyme structure and monitor changes occurring in structural evolution.

In MD simulations of an isolated ND1, the overall global structural changes are already seen in the trajectories of the order of 100 ns (SI Fig. S5); but most prominently the change occurs already in the first 1–3 ns of the trajectories, which indicates the release of the stressed conditions of ND1 subunit in the enzyme structure. The SI provides detailed data that illustrate the overall evolution of the entire ND1 subunit upon release of the restraints. The main qualitative result is shown in SI Fig. S6. It turns out that already in the first few nanoseconds of the trajectory the helices forming the bottleneck are moving, with TM1 straightening up, and AH1 moving up to open the bottleneck. The further evolution results in structures with an overall greater opening of the bottleneck due to movement of the helices involved.

A clear tendency in the expansion of the structure to open the bottleneck is also seen in the low-frequency modes at different time-segments of a long trajectory, see SI Movies S2–S4, with corresponding data in SI Table S1–S4.

Having these insights, we next explore in greater detail how the ND1-surrounding enzyme structure affects the results described in this section.

## Bottleneck in ND1 subunit in the context of the enzyme structure. Coarse-grained simulations

Here we put ND1 subunit in the context of the entire enzyme structure. The question is how the adjacent subunits of ND1 are affecting its fluctuations. As simulations with all-atomic force-field are limited in timescales, here we apply less accurate but more efficient coarse-grained (CG) simulations using Martini force field [36, 37]. These simulations can be expected to yield a reasonable qualitative picture. The simulated system includes all subunits that can directly affect the motion of ND1 structure and include subunits ND1/Nqo8, Nqo6, 4, 9, and A, simulated in the membrane and solvent environment. (One should keep in mind that the bottleneck opening can in principle involve the whole structure of the protein; however, the available structure of the enzyme with the bound quinone, does not show such clear global conformational changes. We, therefore, focused on the local changes—ND1 and surrounding subunits). The simulation details are given in SI MD methods.

Here we first compared the all-atomic simulations of an isolated ND1 subunit with the same simulations using Martini coarse-grained force field. Numerical comparison was done by calculating the overlap of the lowest frequency PCA modes of both force-fields. Not surprisingly, quantitatively the PCA modes are quite different in the two force fields (the overlap is low); however, the qualitative comparison is still possible. Namely, the bottleneck opening in the low-frequency modes is clearly seen in both force-fields simulations and involves the same elements: TM1, AH1, and the loop between then among others. However, the amplitudes of motion of different elements are regulated by the details of the force fields, which are quite different in the two cases. In particular, the coarse-grained Martini involves the phenomenological pair-wise rubber band restraints on the sites (5 kJ/mol/Å [2]) within each subunit, which in the long-term simulation keeps the overall structure as it appears in the initial pdb; but at the same time there is no direct analogy to such terms in the all-atomic force field. We explored different possibilities in the variation of this parameter, recognizing that in any case the results should be taken only as qualitative indication of possible dynamic behavior of the system. We then explored PCA modes of a multi-subunit system.

**Fig. 6:**
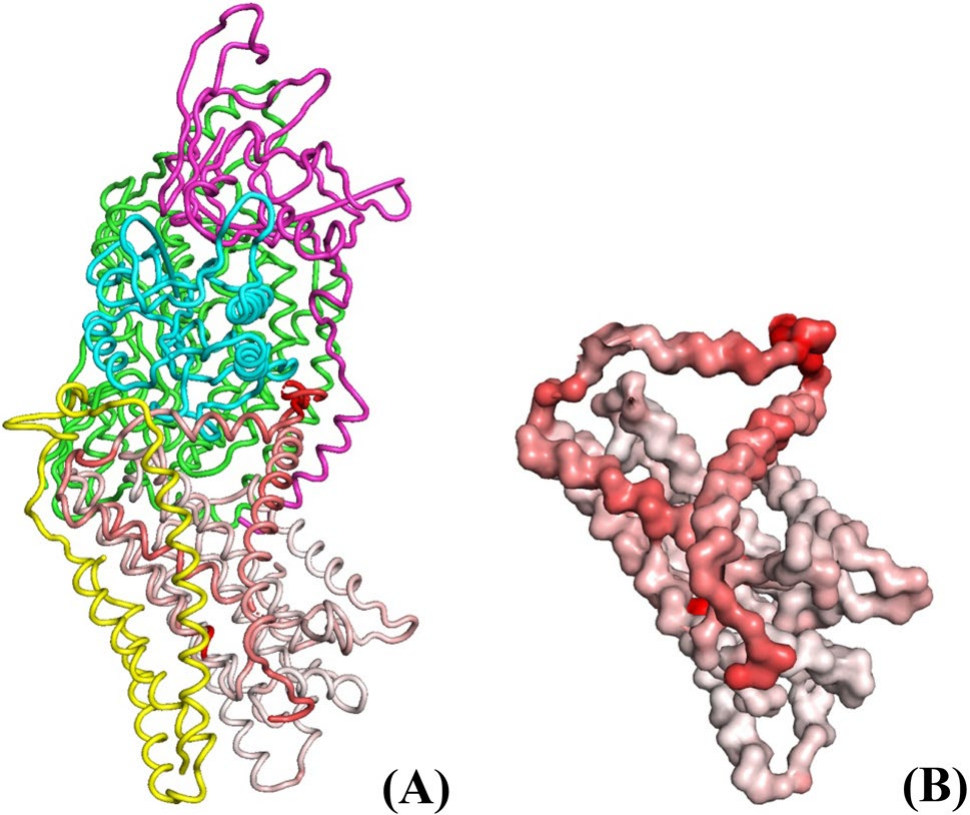
CG PCA lowest-frequency mode of the structure of ND1 (**B**), and (**A**) with additional subunits: A (yellow), 6 (cyan), 4 (green), and 9 (magenta), all shown in (**A**). Red color intensity corresponds to elements with high participation in the Q0 PCA mode. The open structure resulting from the full amplitude Q0 PCA mode is shown

A typical qualitative picture is shown in Fig. 6; it is seen that the lowest frequency PCA mode, as indicated by our participation value rendered in color, mostly involves the structural elements of the bottleneck: TM1, AH1, and TM2, essentially same as in all-atomic simulations. This is interesting and unexpected, at first sight, as neighboring subunits do restrict ND1 motions—but obviously not as much as could be expected. Therefore, these simulations confirm and validate results from the previous section of all-atomic simulations of isolated ND1 with an “effective” artificial environment of ND1.

Overall, these results indicate that thermal fluctuations indeed mostly affect the bottleneck structure, even in the enzyme context, providing the needed opening states along the dynamic trajectory. In the following, we discuss the mechanism of the bottleneck passage that involves these rare fluctuations.

## Discussion and conclusions

### The bottleneck is too narrow for a free passage. The eye of a needle

Here and previously [28], we showed that in five published structures of complex I—bacterial *T. thermophilus*, yeast *Y. lipolytica*, and three mammal, mice, ovine, and human, the bottleneck at the entrance of the quinone chamber is too narrow for a quinol or quinone to pass through it. Most recent additional structures of different conformational states show that the bottleneck in all available structures (twenty-three analyzed so far) is about the same and, therefore, impossible for the quinone to get in or out of the binding cavity unless driven by a conformational changes that presumably occur in the course of thermal fluctuations of the enzyme. This is confirmed by MD simulations of the barrier formed by the narrow entrance in an intact pdb structures. Moreover, the bottleneck appears to be too narrow even for a passage of the isoprenoid tail of ubiquinone in the case of the yeast enzyme, although one quinone molecule is seen as stuck half-way to the binding site in the yeast structure. The shuttle model, where one quinone molecule never gets out the binding cavity appears to be unlikely, as we discussed previously [28].

The conclusion is that fluctuations of the structure not reflected in the pdb structures has to be included in the complete picture. In addition, minor deformations may also result from artificial, out of the membrane conditions [38], both in X-ray or cryo-EM, as the lack of proper membrane solvation of the enzyme [38] can deform the molecule in such a way that the intrinsically narrow entrance path becomes even smaller, impossible for actual passage of quinone and render enzyme appear to be non-functional. To open the entrance of the quinone chamber, some conformational changes are needed; however, the nature of these changes—given their collective character—is not trivial.

Here, using PCA modes of the MD trajectories of the bacterial enzyme, we have identified collective conformational changes that open the quinone chamber bottleneck. The main qualitative result is shown in Fig. 4, and in animation S1 in SI. The changes involve mostly TM1 helix, which straightens up, AH1 helix, which moves up to open the structure, and the loop between them; but in general, the changes involve rearrangement of a larger part of the enzyme. The simulations allow to reconstruct to some extent the elusive structure of ND1 in which the bottleneck is open. It is now clear (given most recent structural data) that the open state is unstable, producing a very low population, which is not readily captured in plunge-freezing of cryo-EM.

### Quantitative estimates of timescales and barriers

The barrier crossing rate by a quinone molecule at the bottleneck can be estimated from the transition state theory; namely, the rate of a single barrier crossing—be it a headgroup, or one of the methyl groups of the isoprenoid tail, is given by the following expression:

$$k \approx \frac{{D_{q} }}{{L_{0}^{2} }}10^{{ - \frac{{V_{b} }}{\ln (10)RT}}}$$

where $D_{q}$ is the diffusion coefficient of quinone in the membrane environment, $D_{q} \sim 10^{ - 7} - 10^{ - 8} {\text{cm}}^{{2}} {\text{/s}}$ [21], and $L_{0}$ is a characteristic length of the barrier width [39]. The first factor (assuming barrier width 1–3 Å) is of the order of 10^8^ s^−1^. The exponential factor should be greater than 10^–5^; as ln10RT = 6 kJ/mol, $V_{b} \approx 30\;{\text{kJ/mol}}$ or less. All our energy barriers are in gross excess of this critical value, thus in the functional state the bottleneck structure should be opened.

Although it is not clear to what extent the structure is opened in the transition state, it appears unlikely that the structure would be open only for a free tail passage while blocking the headgroup. This is because the methyl groups of isoprenoid tail present almost the same challenge of passing the narrow bottleneck as the headgroup, as our calculations suggest. The effective diffusion constant for isoprenoid tail movement through the bottleneck is modified by the same exponential factor discussed above and is too small to be operational in all structures examined. Thus, taken as is in pdb structures, the bottleneck is too narrow to be operational even for the shuttle model [40].

### The squeeze-in model and the kinetic analysis

It is clear that conformational changes are needed to open the bottleneck for the diffusion-like motion of the quinone through the bottleneck; presumably, they occur in thermal fluctuations along the low-frequency PCA modes described above. These fluctuations would allow individual random-walk steps to occur in the overall diffusion-like motion. In such a squeeze-in mechanism, of getting through the eye of a needle, the overall entrance remains to be relatively narrow, which allows for tight control of the entrance of the binding cavity. This could provide desirable selectivity of admission of quinone vs quinol, or vs other bulky molecules by the quinone cavity.

The available kinetic data [25] appear to support this conclusion. Consider the efficiency parameter defined as $\kappa_{BM} = k_{cat} /K_{m}$ for quinone binding and reduction, assuming Michaelis–Menten kinetics. Under the condition of $k_{cat} > > k_{dis}$, the efficiency parameter is the second-order rate constant that can be estimated as follows. (Alternative case $k_{cat} < < k_{dis}$, i.e. the opposite to what we assumed in the above, is an unlikey scenario [28]) In 3D diffusional model [28, 41]

$$\kappa_{BM} = 4\pi r_{0} D_{q} (N_{A} /10^{3} )P_{r}$$

Here the diffusion of quinone head-group from the membrane internal medium to the entrance of Q-tunnel is envisioned as a 3D process, see Fig. 1A. It is assumed that the binding site is a 3D sphere of radius r_0_ (the rate is half of the above for a half-sphere), diffusion coefficient (for center of mass) of substrate is *D*_*q*_, the Avogadro number is introduced for conventional units M^−1^ s^−1^, and CGS units are assumed for *r*_*0*_ and *D*_*q*_. The factor *P*_*r*_ describes the probability that a substrate arriving at the binding site via diffusion will have a right orientation to for binding, and/or that the binding site is open. The more intricate binding site configuration is, the smaller the probability *P*_*r*_; this could be combined with the *effective* capture radius, *r*_*0*._ However, the remaining part of probability *P*_*r*_ is the probability that the binding site is open.

An equivalent expression for 2D diffusion [42, 43] in the membrane plane gives qualitatively similar results. Here,

$$\kappa_{BM} = \frac{{\pi D_{q} d_{m} (N_{A} /10^{3} )P_{r} }}{{\ln (R_{0} /r_{0} )}}$$

where *d*_*m*_ is the membrane width (of the order of 50 Å), and *R*_0_ is the typical distance between substrate molecules in the membrane (for [*Q*] = 10 mM, *R*_0_ = 10 nm). The factor in denominator is never too large, and realistically is in the range of 5–7 for realistic *r*_0_ = 1 Å, or somewhat less, assuming order of magnitude values. Given these values, the two expressions give qualitatively similar results for *r*_0_ = 1 Å, and the expression is not sensitive to this parameter.

The diffusion coefficient *D*_*q*_ is assumed to be in the range [21, 41] of 10^–7^ to 10^–8^ cm^2^/s for the substrates of our interest, but can be modified by the barrier at the bottleneck, as discussed earlier.

The above expressions predict rates that are in line with the available kinetic data. For example, for NADH oxidation reaction of Ref. [25] both theory and experiment give *k*_*cat*_*/K*_*m*_ = $\kappa_{BM} \sim$ 10^7^, for *r*_*0*_ = 1 Å and *P*_*r*_ = 1 (no need to open the binding site). Similar values are obtained for AOX of Ref. [25].

However, for Q-reduction *k*_*cat*_*/K*_*m*_ in Ref. [25] is much smaller and, depending on the length of isoprenoid tail, is in the range of 10^4^ to 10^5^. This significant reduction can be readily explained by an additional small factor *P*_*r*_ in the range of 10^–2^ to 10^–3^ or even smaller (effective reduction of the capture radius, *r*_*0*_, would not produce such an effect). This could be rationalized by the difficulty of passing through a narrow entrance of the Q-channel and be interpreted as a small probability of the bottleneck open state.

At the same time, the difficulty of quinone accessing the reduction catalytic site and the relatively fast reduction of the FeS chain by NADH (that provide electrons for quinone reduction) should result in a (partially) reduced state of FeS clusters in the chain, which then can serve as a buffer of electrons reducing quinone. In such conditions, the redox potential of the FeS chain would be in equilibrium with that of NADH pool, i.e. around -320 mV, and thus reduction of quinone to produce semiquinone state appears to be quite possible, despite that the redox potential of the last FeS cluster in the chain N2, which reduces quinone, by itself is much more positive [24].

## Supplementary Information

Below is the link to the electronic supplementary material.

Supplementary file1 (PDF 889 KB)

Supplementary file2 (MP4 14819 KB)

Supplementary file3 (MP4 14819 KB)

Supplementary file4 (MP4 14819 KB)

Supplementary file5 (MP4 14819 KB)

Supplementary file6 (MP4 14819 KB)

Supplementary file7 (MP4 14819 KB)

Supplementary file8 (MP4 14819 KB)

Supplementary file9 (MP4 14819 KB)

Supplementary file10 (MP4 14819 KB)

Supplementary file11 (MP4 14819 KB)

Supplementary file12 (MP4 14819 KB)
