# Supplementary material for: Quinone binding in respiratory complex I: Going through the eye of a needle. The squeeze-in mechanism of passing the narrow entrance of the quinone site
Source: Photochem Photobiol Sci. 2021 Nov 23;21(1):1–12. doi: 10.1007/s43630-021-00113-y (PMC8799541; doi:10.1007/s43630-021-00113-y)
Supplement: Supplementary file 1 — Supplementary file1 (PDF 889 KB) [file 43630_2021_113_MOESM1_ESM.pdf]

Supplementary Information

Quinone binding in respiratory complex I: The squeeze-in model for passing the entrance of the quinone site

Nithin Dhananjayan, Panyue Wang, Igor Leontyev, and Alexei A. Stuchebrukhov*

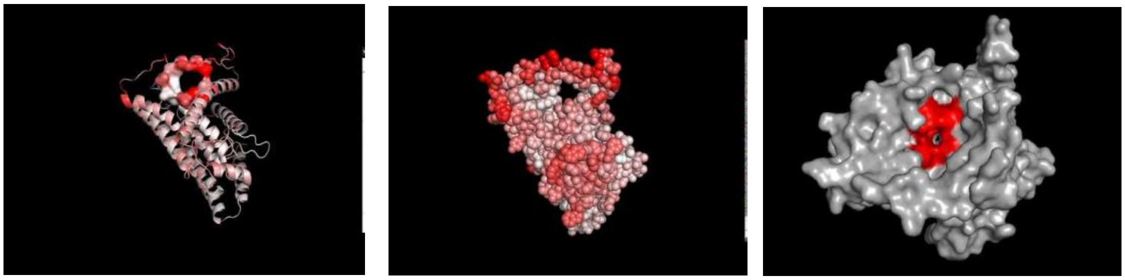

Movie S1. Left, the helix structure representation of H-subunit (ND1) of complex 1 and the entrance into the Q-binding cavity. Middle, same in all atom representation. The opening of the entrance bottleneck occurs in deformation along the lowest frequency collective PCA mode (Q0). The red color represents residues that contribute most to PCA mode Q0. The un-bending deformation of TM1 helix and the increased size of the bottleneck are two most prominent features of Q0. Center, same in all atom-representation. Right, deformation of E-channel along Q0. Note: To play video double click on the icon, the video opens in YouTube. To loop the YouTube video: if on PC right-click and choose “Loop” to loop the video; if on MAC, click (Control^+one mouse click).

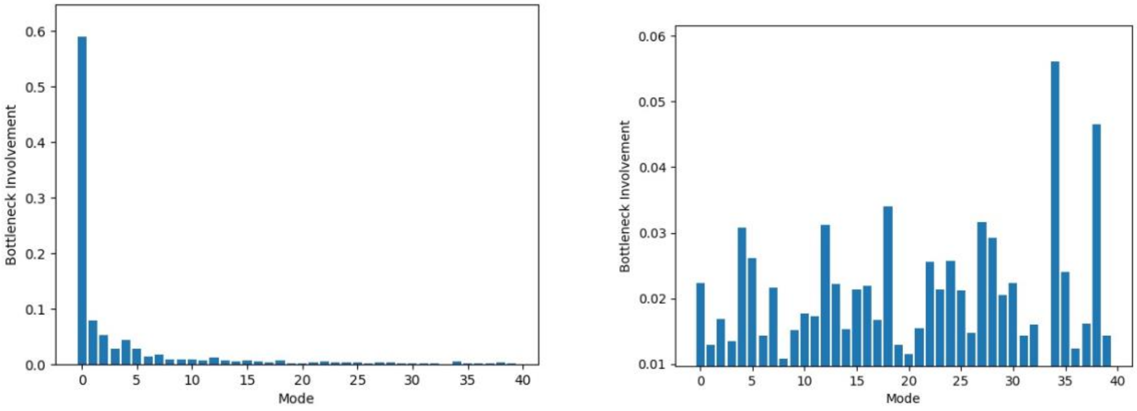

Figure S1. Left: Amplitude-scaled and normalized bottleneck involvement of PCA modes; Right: same without amplitude (eigenvalue) scaling and normalization – shown for better resolution of mode contribution. Spectrum of bottleneck involvement over first 40 modes of the

1ns run. To find the involvement of the bottleneck residues, A29, F28, P59, D62, A63, S66, I239, A242, L243, and M246, we calculate $I_\lambda = \sum_{a \epsilon \text{bottleneck}} P_{a\lambda}$. Then these values are amplitude(eigenvalue)-scaled and normalized. Higher modes are increasingly stiffer with less relevance to large scale structural changes. Mode 0, 4 and 5 show a higher bottleneck involvement than the rest of the first 10 modes, which indicates more prominent configuration changes of the bottleneck. These modes are shown in animation/Movies S1.

Table S1. First ten PCA modes characteristics of a 1ns MD trajectory.

mode	eigenvalues (A^2)	spring constants (KJ/mol/A^2)	effective masses (g/mol)	angular frequencies (rads/s)	frequencies (Hz)	periods (s)
0	2.11E+03	1.16E-03	108.77	3.26E+10	5.19E+09	1.93E-10
1	4.87E+02	5.01E-03	110.60	6.73E+10	1.07E+10	9.34E-11
2	2.49E+02	9.78E-03	111.53	9.36E+10	1.49E+10	6.71E-11
3	1.65E+02	1.48E-02	109.31	1.16E+11	1.85E+10	5.40E-11
4	1.14E+02	2.15E-02	112.44	1.38E+11	2.20E+10	4.55E-11
5	8.71E+01	2.80E-02	113.14	1.57E+11	2.50E+10	4.00E-11
6	7.83E+01	3.11E-02	112.35	1.66E+11	2.65E+10	3.78E-11
7	6.58E+01	3.70E-02	110.71	1.83E+11	2.91E+10	3.44E-11
8	6.12E+01	3.98E-02	110.55	1.90E+11	3.02E+10	3.31E-11
9	4.76E+01	5.12E-02	111.07	2.15E+11	3.42E+10	2.93E-11

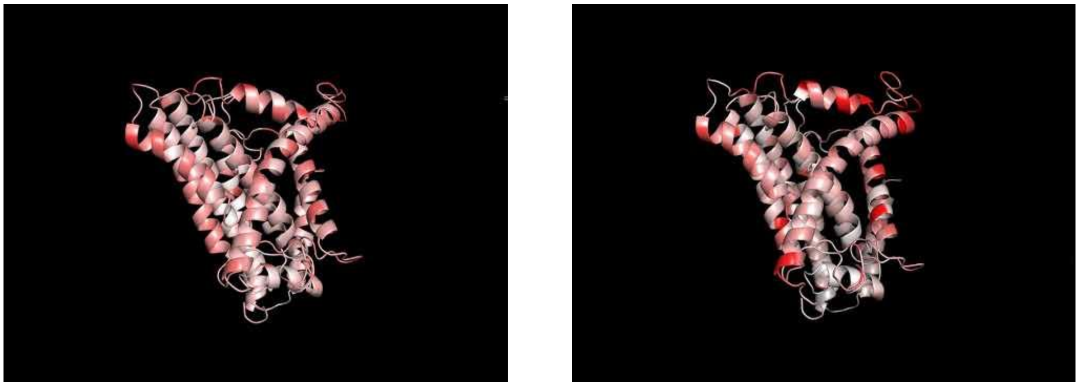

Movie S1ab. Mode 4 and Mode 5 of the 1ns run.

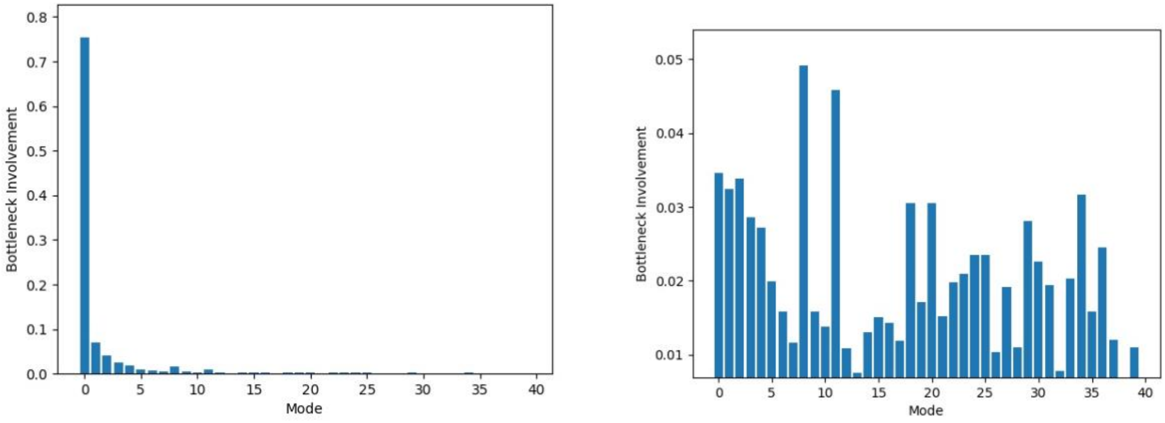

Figure S2. Similar to Fig. S1. Spectrum of bottleneck involvement over first 40 modes of the 3ns run. Mode 0, 8 and 11 show a higher bottleneck involvement than the rest of the first 40 modes, which are shown in animation/Movie S2 respectively.

Table S2. Computer output of the 3ns MD run. The periods of the modes show remarkable similarity to Table S1. Notice about same masses, as for mass-weighted coordinates (see text).

mode	eigenvalues (A^2)	spring constants (KJ/mol/A^2)	effective masses (g/mol)	angular frequencies (rads/s)	frequencies (Hz)	periods (s)
0	4.41E+03	5.53E-04	106.78	2.27E+10	3.62E+09	2.76E-10
1	4.44E+02	5.49E-03	113.84	6.94E+10	1.11E+10	9.05E-11
2	2.43E+02	1.00E-02	108.45	9.61E+10	1.53E+10	6.54E-11
3	1.78E+02	1.37E-02	114.10	1.09E+11	1.74E+10	5.74E-11
4	1.37E+02	1.78E-02	110.59	1.27E+11	2.02E+10	4.95E-11
5	9.61E+01	2.53E-02	114.22	1.49E+11	2.37E+10	4.22E-11
6	8.00E+01	3.04E-02	114.90	1.63E+11	2.59E+10	3.86E-11
7	7.77E+01	3.14E-02	111.40	1.68E+11	2.67E+10	3.74E-11
8	6.80E+01	3.59E-02	118.01	1.74E+11	2.77E+10	3.60E-11
9	4.92E+01	4.95E-02	114.62	2.08E+11	3.31E+10	3.02E-11

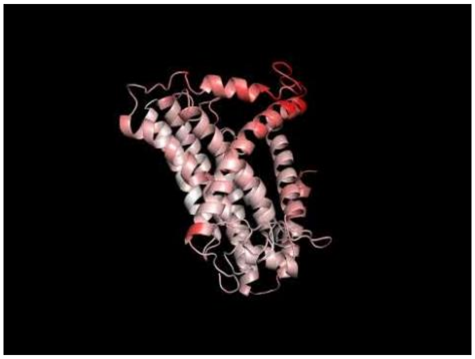

Movie S2a. Mode 0 of the 3ns run.

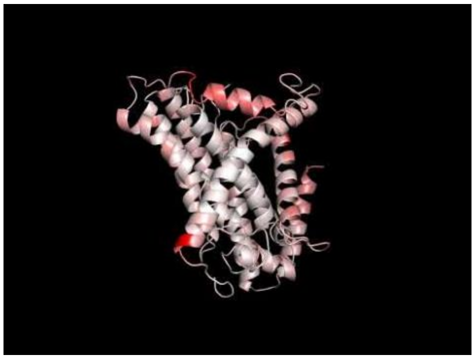

Movie S2b. Mode 8 of the 3ns run.

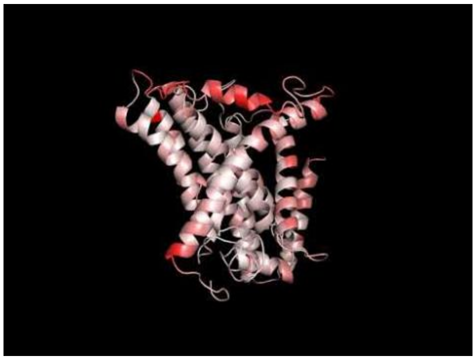

Movie S2c. Mode 11 of the 3ns run.

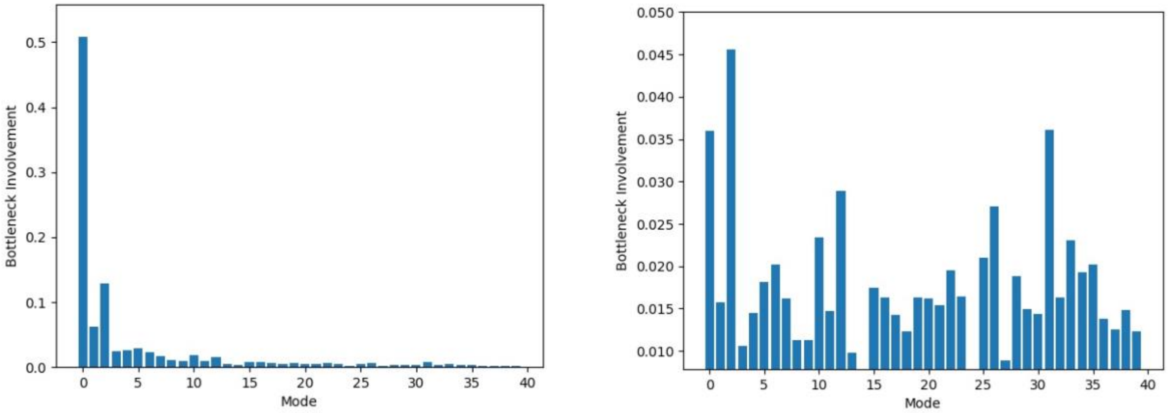

Figure S3. Spectrum of bottleneck involvement over first 40 modes of the 20-30ns window of the 60ns run. Mode 0 and 2 show a higher bottleneck involvement than the rest of the first 40 modes, which are shown in Movie S4a and S4b respectively.

Table S3. PCA analysis of the 20-30ns window of the 60ns MD run. The periods are close to the periods of the 1ns and 3ns runs.

mode	eigenvalues (A^2)	spring constants (KJ/mol/A^2)	effective masses (g/mol)	angular frequencies (rads/s)	frequencies (Hz)	periods (s)
0	8.31E+02	2.93E-03	108.26	5.21E+10	8.29E+09	1.21E-10
1	2.35E+02	1.04E-02	116.43	9.44E+10	1.50E+10	6.66E-11
2	1.66E+02	1.47E-02	110.05	1.15E+11	1.84E+10	5.44E-11
3	1.36E+02	1.79E-02	109.55	1.28E+11	2.03E+10	4.92E-11
4	1.05E+02	2.33E-02	112.38	1.44E+11	2.29E+10	4.36E-11
5	9.23E+01	2.64E-02	114.85	1.52E+11	2.41E+10	4.14E-11
6	6.58E+01	3.70E-02	119.18	1.76E+11	2.81E+10	3.56E-11
7	6.01E+01	4.06E-02	111.73	1.91E+11	3.03E+10	3.30E-11
8	5.69E+01	4.28E-02	122.40	1.87E+11	2.98E+10	3.36E-11
9	5.09E+01	4.79E-02	113.74	2.05E+11	3.27E+10	3.06E-11

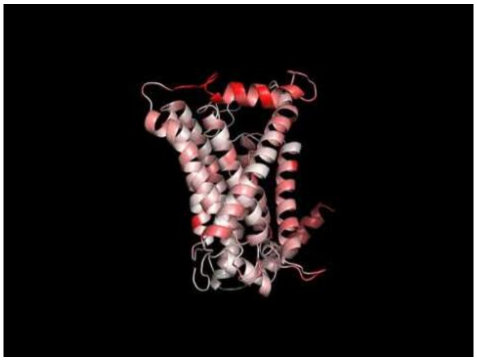

Movie S3a. Mode 0 of the 20-30ns window of the 60ns MD run.

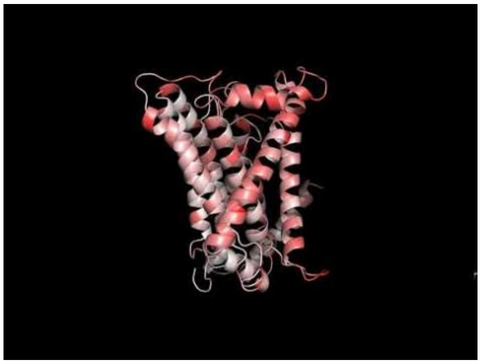

Movie S3b. Mode 2 of the 20-30ns window of the 60ns MD run.

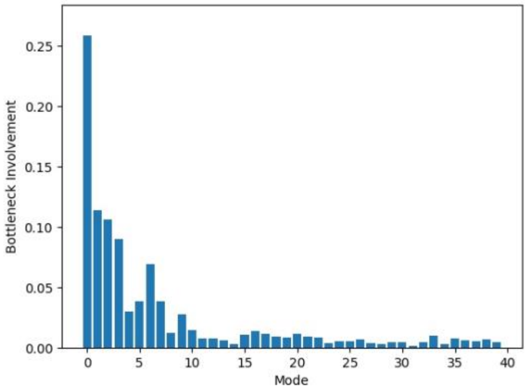

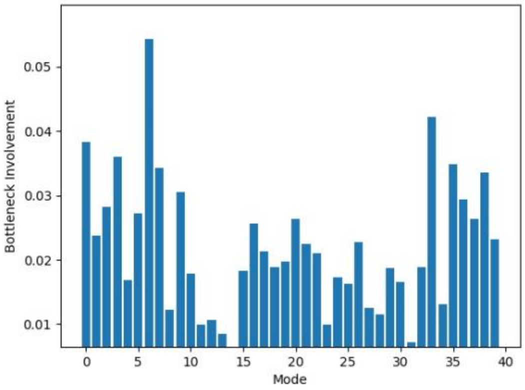

Figure S4. Similar to Fig. S1. Spectrum of bottleneck involvement over first 40 modes of the 50-60ns window of the 60ns run. Mode 0 shows a relatively higher bottleneck involvement, which is illustrated in animation/Movie S4.

Table S4. PCA analysis of the 50-60ns window of the 60ns MD run. The periods are close to the periods of the 1ns and 3ns runs. There is a gradual decline in eigenvalues across the first 10 modes comparing to the 20-30ns PCA analysis shown in Table S3.

mode	eigenvalues (A^2)	spring constants (KJ/mol/A^2)	effective masses (g/mol)	angular frequencies (rads/s)	frequencies (Hz)	periods (s)
0	2.69E+02	9.07E-03	111.87	9.00E+10	1.43E+10	6.98E-11
1	1.92E+02	1.27E-02	111.19	1.07E+11	1.70E+10	5.88E-11
2	1.49E+02	1.63E-02	116.04	1.19E+11	1.89E+10	5.30E-11
3	1.00E+02	2.44E-02	112.48	1.47E+11	2.34E+10	4.27E-11
4	7.07E+01	3.45E-02	113.26	1.74E+11	2.78E+10	3.60E-11
5	5.60E+01	4.35E-02	113.62	1.96E+11	3.11E+10	3.21E-11
6	5.08E+01	4.79E-02	115.33	2.04E+11	3.24E+10	3.08E-11
7	4.48E+01	5.44E-02	111.48	2.21E+11	3.51E+10	2.85E-11
8	4.06E+01	6.01E-02	114.56	2.29E+11	3.64E+10	2.74E-11
9	3.67E+01	6.64E-02	117.52	2.38E+11	3.78E+10	2.64E-11

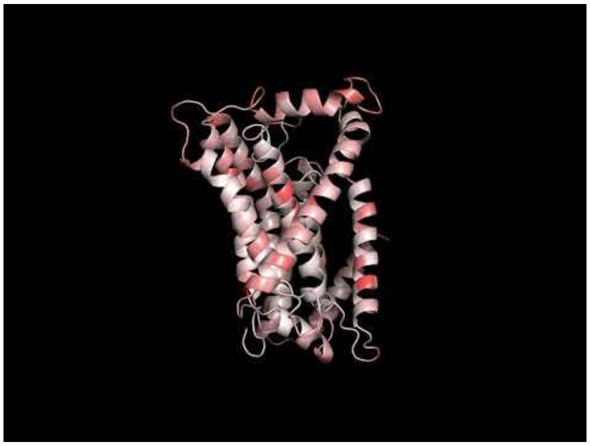

Movie S4. Mode 0 of the 50-60ns window of the 60ns MD run.

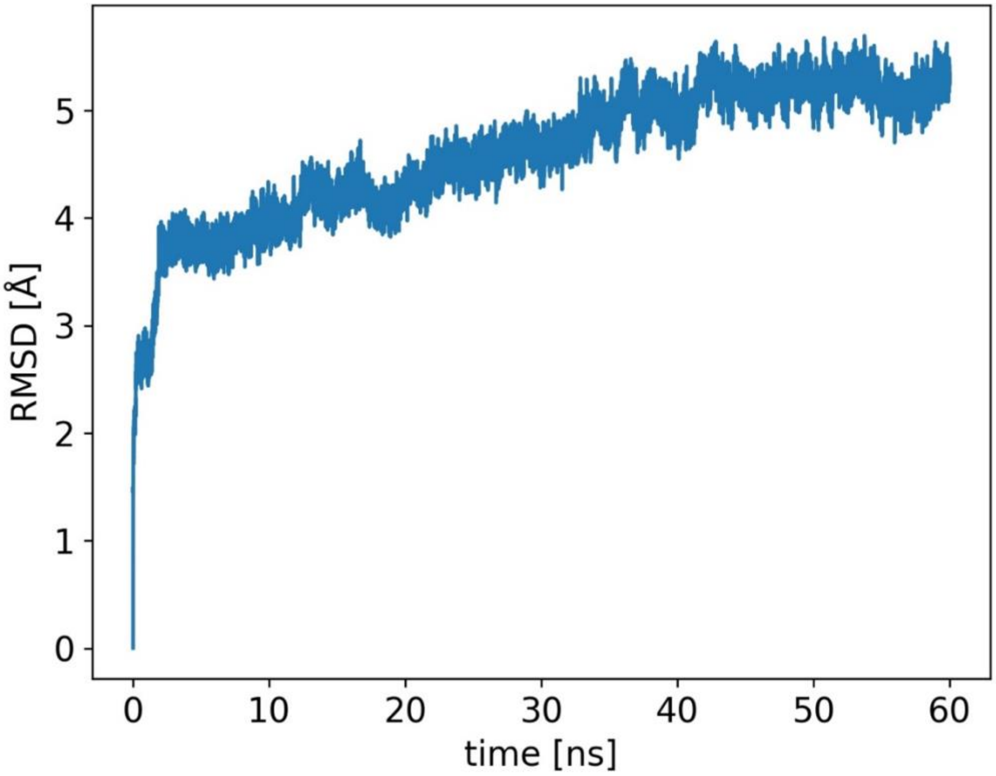

Figure S5. Overall structure expansion of ND1. Shown is the root-mean-square deviation (RMSD) in a 60ns MD simulation of chain H. From the starting point to 1ns, the RMSD increased from 0 Å to 2.6 Å. Another sharp increase happens from 3ns to 4ns, where RMSD increased from 2.6 Å to 3.7 Å. Then the RMSD slowly grows from 3.7 Å to 4.7 Å during the 10ns to 60ns period. From 10 to 40 ns, the drift is mainly due to drift in the center of mass, which was fixed in the segment 40 to 60ns. The PCA modes are adjusted along the trajectory as shown in the above data, S1-S4.

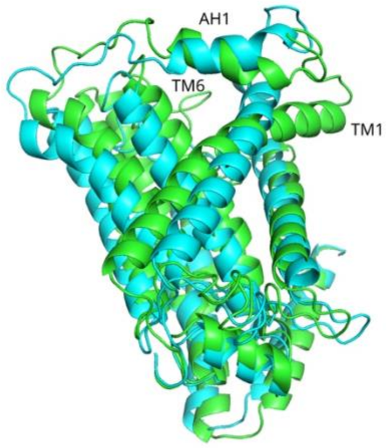

Figure S6a. Details of the expansion. Crystal structure (green) vs. structure after 1ns (cyan) of MD simulation. TM1 straightens up as AH1 bends upwards. TM2 and TM6 shift to the left.

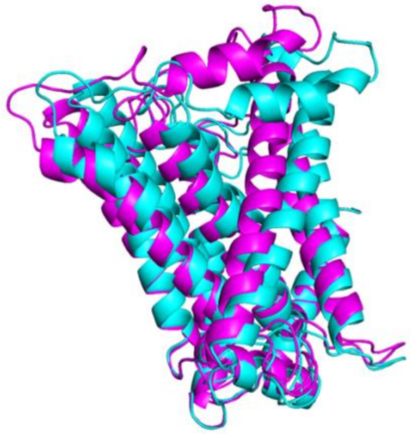

Figure S6b. structure after 1ns (cyan) vs. structure after 20ns (magenta) of MD simulation. AH1 continues to shift upward while TM1 shifts to the left. TM2 and TM6 slightly move to the left.

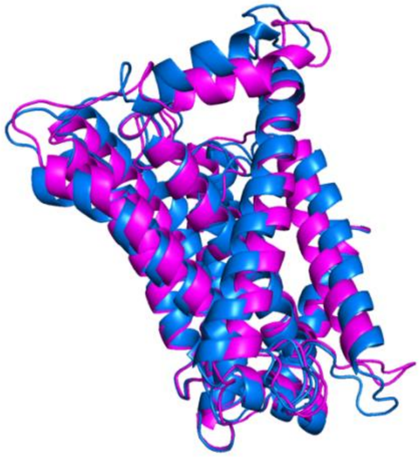

Figure S6c. structure after 20ns (magenta) vs structure after 50ns (navy blue) of MD simulation. AH1 continues to move upwards. TM1, TM2 and TM6 remain relatively stationary comparing to AH1.

Methods: MD Simulations

Coarse grained simulations

The Coarse Grained (CG) simulations were performed using Martini force field [1-3] and GROMACS simulation package. Setting up the coarse-grained protein simulation was done by converting the atomistic protein structure (pdb code 4HEA) into a coarse-grained model and generating suitable Martini topology by python script "martinize.py" (part of the Martini tool kit). The insertion of the protein into lipid membrane and solvation in water was done by another Martini tool "insane.py" and GROMACS utility "solvate". Our complex-I model includes subunits 4, 5, 6, 9, W, A and H. The iron-sulfur clusters were converted to a single sphere with a charge of -2 (oxidized state).

Keeping in line with the overall Martini philosophy, the coarse-grained protein model groups 4 heavy atoms together in one coarse-grained bead. Each residue has one backbone bead and zero to four side-chain beads depending on the residue type. In addition to bead representation of amino acids in protein simulations Martini Method implies application of elastic networks (distance restrains between certain multiple backbone beads within the same subunit). These restraints preserve the protein secondary structure from unfolding due to approximate nature of interactions in the coarse-grained force field. The elastic network restrains, however, are not applied between subunits, i.e. mutual dynamics of subunits in the complex-I is free. This standard for protein simulations Martini protocol is used in the present work unless otherwise is explicitly stated. We also performed one PCA test for the model in which some elastic network restraints were removed within subunit H in order to study the effect of conformational changes that involve changes in the secondary structure. More specifically in this test the elastic network restraints were removed between loops and helices AH1, TM1, TM2, TM6 and all within TM1.

In the CG simulations the time step 20 fs (which is a part of the CG force field) was used. The van der Waals interactions are smoothly shifted to zero between 0.9 and 1.2 nm, adhering to the standard Martini protocol. The electrostatic interactions are treated by the reaction-field method with the dielectric constant $\varepsilon_r = 15$ and are smoothly shifted to zero between 0.0 and 1.2 nm as recommended in ref [4]. The neighbor list of 1.4 nm is updated every 10 steps. Pressure (1 bar) and temperature (298.15 K) coupling parameters, τ_t and τ_p, are set to 1.0 and 12.0 ps. The length of simulations for different runs is specified explicitly for each test and varies from 10 to 100 μs.

All-atomic Bottleneck Opening simulations

Here we use GROMACS[5] simulation package with CHARMM36[6] forcefield. The initial coordinates of H-subunit were extracted from the whole structure 4HEA of *T. thermophilus*[7]; all charged residues were neutralized to emulate counter-ions of the solvent and those of the salt-bridges (indeed most charged residues of ND1 have salt-bridge counter-ions; however, different charge-schemes were also explored with similar qualitative results), and the structure was placed in a simulation box filled with artificial hydrophobic medium of Lennard-Jones spheres at ambient temperature of 310 Kelvin. The medium was also modeled using tip3p water, with the same qualitative results for PCA modes. The solvation of structure was then energy minimized with all H-subunit atoms restrained (with 5000 kJ/mol/nm^2 force constant) to preserve the original structure. The structure was then simulated under periodic boundary conditions and stochastic dynamics[8, 9] to maintain temperature 310 Kelvin. The time step was 1fs, and τ_t was 2 ps. The Canonical ensemble (NVT) is used in these simulations. In the NVT simulations, only the terminal residues of H-subunit - 2, 3, 4, 5, 352, 353, and 354 were restrained with 500kJ/mol/nm^2 force constants and all other atoms were free. The van der Waals interactions were treated with Verlet [10] cut-off scheme with Verlet buffer tolerance of 0.005 KJ/mol/ps per atom and van der Waals cut off of 1.2 nm. The electrostatics were handled by Particle-Mesh Ewald[11] with a Fourier spacing of 0.12 nm and a PME order of 4.

Up to 100ns trajectories were generated using the methods above. (The longer trajectories were not needed in our study.) The RMSD for a trajectory was generated using the GROMACS rmsd utility. The center of mass rotation and translation were removed.

Pulling simulations

UQ/DQ/MQ Models:

The ubiquinone model UQ10 was generated using Swiss Param. The DQ/MQ and shorter tail and reduced analogues were all based on this model. To create UQ1 and UQ3 models, the UQ10 model was shortened by cutting of the tails to have either 1 or 3 isoprenoids, and a hydrogen placed where the tail was cut. The reduced models were created by taking the oxidized models and converting the ketones to enols in the headgroups. Specifically, hydrogens were bonded to the oxygens with bond length 0.096nm and strength of other single bonds in the forcefield, a bond angle of 108 degrees and force constant of 543.920 KJ/mol/rad^2. For each hydrogen, 2 proper dihedral angles (corresponding to the 2 carbons connected to the enol carbon) were added with dihedral angles of 180 degrees (trans), force constant of 4.142 KJ/mol and multiplicity of 2. Carbon-carbon single and double bonds in the ring of the head group were then shifted to match the standard ubiquinol structure using existing carbon-carbon single and double bond parameters in the headgroup. The MQ models were created by taking the corresponding UQ models, removing the methoxy groups and adding a ring corresponding to the head group of the standard menaquinone structure using the parameters that already existed in the model for similar bonds, angles, and dihedrals. The DQ models were made from the corresponding UQ models, replacing the isoprenoid tails with a simple saturated hydrocarbon chain, once again using the parameters that already existed in the model for corresponding bonds, angles, and dihedrals.

Simulations within the membrane:

The structure was constructed using charm GUI[12-14] with using 4HEA (keeping chain D, F, G, I and P), POPC lipid (315 upper leaflet, 300 lower leaflet). Pre-MD equilibration was done using energy minimization, followed by NVT equilibration at 310 Kelvin, then NPT equilibration at 310 Kelvin and 1 bar. The Berendsen[15] thermostat and the Berendsen barostat were used to maintain temperature and pressure. These were maintained while pulling the quinone through the bottleneck. The Coulomb and LJ interactions have a cutoff radius of 1.2 nm.

Three trajectories were generated using GROMACS 2020. The pulling rate of the three trajectories are 0.01 nm/ps, 0.01 nm/ps and 0.02 nm/ps respectively. The simulation time are 300 ps, 300 ps and 180 ps respectively. The pulling force constant for all three trajectories is 2500 kJ/mol/nm^2.

Simulations with hydrophobic medium:

The models of bottlenecks were taken from the crystal structures and neutral charge states chosen for all residues so that the bottleneck had zero net charge. The bottleneck models were placed into artificial hydrophobic media of LJ spheres, equivalent to tip3p water with all charges set to zero. The system was then energy minimized with the bottleneck model positions restrained with 2500 KJ/mol/nm^2. The pulling simulations were then done with NVT using stochastic dynamics at 310 Kelvin.

Ligand	Bottleneck	Sim. Length	# runs
UQ1	t. Thermophilus	1.5 ns	53
UQ1H2	t. Thermophilus	1.5 ns	59
UQ3-tail	t. Thermophilus	1 ns	29
UQ3	ovine	1.5 ns	15
UQ3	human	2 ns	15
UQ3	y. Lipolytica	2 ns	15
UQ3H2	y. Lipolytica	2 ns	15

MQ1	t. Thermophilus	1.5 ns	31
MQ1H2	t. Thermophilus	1.5 ns	30
DQ1	t. Thermophilus	1.5 ns	31
DQ1H2	t. Thermophilus	1.5 ns	31

Table S5: List of all pulling simulations through simplified bottleneck in hydrophobic media. Data shown to demonstrate statistical averaging over trajectories used in free energy calculations.

Barrier simulations

The MD simulation details are the same as in Ref.[28] of main text. Briefly, the protein was incorporated into POPC membrane. The quinone was placed near the entrance of Complex I, and after equilibration, was pulled into and then out of the quinone cavity. The energy and the pulling force were measured along the pulling trajectory. Both ubiquinone and menaquinone with various tail lengths were simulated, see details in Ref.[28] of main text.

To improve statistics, focused MD simulations on a restricted system that involved only the residues of the bottleneck (Fig. 1 of the main text) were used. In this case the simulation box was filled with an artificial hydrophobic (LJ) medium. Typically, Q was pulled with a speed of 0.1 Å per ps; this is about ten times slower than the rate of water molecule diffusion in bulk water (D_w=10^{-5} cm^2/s)[30]. In a focused MD the pulling speed was further reduced by a factor of ten to simulate adiabatic conditions for free energy evaluation.

Thermodynamic work to cross the bottleneck

Using pulling trajectories, we calculated the Helmholtz work (or Helmholtz free energy, W) to cross the bottleneck. The work is defined by the average force needed to pull the quinone through the bottleneck along the pulling coordinate l:

$$\left(\frac{\partial W}{\partial l}\right)_T = \langle F \rangle$$
$$\Delta W = \int_{(i)}^{(f)} \langle F \rangle d\,l \tag{0.1}$$

The above relations assume a reversible or quasi-equilibrium (in practice very slow) change of the system. For this reason, the pulling rate was set to 0.01 Å per ps, which is about hundred times slower than the diffusion rate of water. The force $\langle F \rangle$ is assumed to be averaged over thermal fluctuations (for a given l, and over a set of initial conditions in the initial state (i)). In our calculations, the pulling force $F(t)$ is measured along the MD pulling trajectory and averaged locally (moving average) over a short interval of 100 ps, for a typical pulling trajectory of some 1500ps. In addition, the averaging was done over some 30-60 pulling trajectories with different initial conditions. The Helmholtz work ΔW gives the energy barrier along the trajectory.

When the final state of quinone (*f*) is defined as a region at the top of the barrier, i.e. the position of the quinone head group inside the bottleneck, and the initial state (*i*) is a region inside the Q-chamber, the work described above is related to both decrease of entropy (as in isothermal contraction of gas) and

increase of potential (elastic) energy of the system due to possible structure adjustment of putting the oversized quinone head group into a relatively narrow bottleneck of the cavity entrance.

In addition, we calculated so-called Jarzynski Averaged (JA) work[31],

$$\left\langle \Delta W \right\rangle_J = -RT \ln < e^{-\frac{\Delta w}{RT}} > \tag{0.2}$$

where Δw is the work for a given pulling trajectory, and the averaging (indicated by the brackets) is assumed over different trajectories. If the pulling work were calculated for a non-equilibrium process at constant energy, instead of constant temperature and quasi-equilibrium process considered here, the Jarzynski equality (JE) would give the proper free energy change. Here the conditions are different from what is assumed in the JE, and thus the above relation is not directly applicable; however, we found it useful to consider this type of averaging as it emphasizes trajectories with minimal work and thus selects (or filter out) the "optimal" trajectories. JA is sensitive to qualitative changes along the trajectory, such as the entrance of the headgroup into the bottleneck or the passage of individual methyl groups of isoprenoid tail of ubiquinone, which show up as small bumps on the JA curves, see Fig. 3 in the main text.

1. Marrink, S.J., et al., *The MARTINI force field: coarse grained model for biomolecular simulations.* J Phys Chem B, 2007. **111**(27): p. 7812-24.
2. Marrink, S.J.F., M.; Risselada, H. J.; Periole, X. , *In Coarse Graining of Condensed Phase and Biomolecular Systems*, in *The MARTINI Force Field*, G.A. Voth, Editor. 2009, CRC Press , 2009: Boca Raton, FL.
3. Periole, X.M., S. J., *The Martini Coarse-Grained Force Field*, in *Biomolecular Simulations: Methods and Protocols*, E.S. Luca Monticelli, Editor. 2013, Springer International Publishing.
4. de Jong, D.H., et al., *Martini straight: Boosting performance using a shorter cutoff and GPUs.* Computer Physics Communications, 2016. **199**: p. 1-7.
5. Abraham, M.J., et al., *GROMACS: High performance molecular simulations through multi-level parallelism from laptops to supercomputers.* SoftwareX, 2015. **1-2**: p. 19-25.
6. Huang, J. and A.D. MacKerell, Jr., *CHARMM36 all-atom additive protein force field: validation based on comparison to NMR data.* J Comput Chem, 2013. **34**(25): p. 2135-45.
7. Baradaran, R., et al., *Crystal structure of the entire respiratory complex I.* Nature, 2013. **494**(7438): p. 443-8.
8. Van Gunsteren, W.F. and H.J.C. Berendsen, *A Leap-frog Algorithm for Stochastic Dynamics.* Molecular Simulation, 2007. **1**(3): p. 173-185.
9. Goga, N., et al., *Efficient Algorithms for Langevin and DPD Dynamics.* J Chem Theory Comput, 2012. **8**(10): p. 3637-49.
10. Páll, S. and B. Hess, *A flexible algorithm for calculating pair interactions on SIMD architectures.* Computer Physics Communications, 2013. **184**(12): p. 2641-2650.
11. Darden, T.a.Y., Darrin and Pedersen,Lee *Particle mesh Ewald: An N·log(N) method for Ewald sums in large systems.* The Journal of Chemical Physics, 1993. **98**: p. 10089-10092.
12. Jo, S., et al., *CHARMM-GUI: a web-based graphical user interface for CHARMM.* J Comput Chem, 2008. **29**(11): p. 1859-65.
13. Brooks, B.R., et al., *CHARMM: the biomolecular simulation program.* J Comput Chem, 2009. **30**(10): p. 1545-614.

14. Lee, J., et al., *CHARMM-GUI Input Generator for NAMD, GROMACS, AMBER, OpenMM, and CHARMM/OpenMM Simulations Using the CHARMM36 Additive Force Field.* J Chem Theory Comput, 2016. **12**(1): p. 405-13.
15. Berendsen, H.J.C., et al., *Molecular dynamics with coupling to an external bath.* The Journal of Chemical Physics, 1984. **81**(8): p. 3684-3690.
